# Effects of multidisciplinary inpatient rehabilitation on everyday life physical activity and gait in patients with multiple sclerosis

**DOI:** 10.1186/s12984-024-01383-0

**Published:** 2024-05-28

**Authors:** Gaëlle Prigent, Kamiar Aminian, Roman Rudolf Gonzenbach, Roger April, Anisoara Paraschiv-Ionescu

**Affiliations:** 1 Laboratory of Movement Analysis and Measurement, École Polytechnique Fédérale de Lausanne (EPFL), Lausanne, Switzerland; 2 Department of Neurological Rehabilitation, Rehabilitation Centre Valens, Valens, Switzerland

## Abstract

**Background:**

Multiple sclerosis is a progressive neurological disease that affects the central nervous system, resulting in various symptoms. Among these, impaired mobility and fatigue stand out as the most prevalent. The progressive worsening of symptoms adversely alters quality of life, social interactions and participation in activities of daily living. The main objective of this study is to bring new insights into the impact of a multidisciplinary inpatient rehabilitation on supervised walking tests, physical activity (PA) behavior and everyday gait patterns.

**Methods:**

A total of 52 patients, diagnosed with multiple sclerosis, were evaluated before and after 3 weeks of inpatient rehabilitation. Each measurement period consisted of clinical assessments and 7 days home monitoring using foot-mounted sensors. In addition, we considered two subgroups based on the Expanded Disability Status Scale (EDSS) scores: ‘mild’ (EDSS < 5) and ‘severe’ (EDSS ≥ 5) disability levels.

**Results:**

Significant improvements in fatigue, quality of life and perceived mobility were reported. In addition, walking capacity, as assessed by the 10-m walking test, two-minute walk test and timed-up-and-go test, improved significantly after rehabilitation. Regarding the home assessment, mildly disabled patients significantly increased their locomotion per day and complexity of daily PA pattern after rehabilitation, while severely disabled patients did not significantly change. There were distinct and significant differences in gait metrics (i.e., gait speed, stride length, cadence) between mildly and severely disabled patients, but the statistical models did not show a significant overall rehabilitation effect on these gait metrics.

**Conclusion:**

Inpatient rehabilitation showed beneficial effects on self-reported mobility, self-rated health questionnaires, and walking capacity in both mildly and severely disabled patients. However, these improvements do not necessarily translate to home performance in severely disabled patients, or only marginally in mildly disabled patients. Motivational and behavioral factors should also be considered and incorporated into treatment strategies.

**Supplementary Information:**

The online version contains supplementary material available at 10.1186/s12984-024-01383-0.

## Introduction

Multiple sclerosis (MS) is a progressive neurological disease that affects the central nervous system, resulting to various symptoms. Among these, impaired mobility and fatigue stand out as the most prevalent in people with MS (pwMS) [8, 27, 50]. The progressive worsening of symptoms adversely alters quality of life, social interactions and participation in activities of daily living (ADLs) [12, 25, 46, 62]. Nowadays, there is no curative treatment for MS. However, inpatient rehabilitation can improve the ability to walk by addressing the problem with a variety of approaches such as strengthening leg muscles, improving balance, increasing cardio-pulmonary fitness, adapting walking aids, reducing fatigue and cognitive deficits, or optimizing medical treatment. In particular, exercise training demonstrated positive effects on muscle strength, mobility functions and aerobic capacity, improving balance, gait and quality of life [34, 38, 51].

The scientific evidences for the effectiveness of inpatient rehabilitation is usually based on either questionnaires or clinical functional assessments. Several walking tests have been developed to evaluate mobility (e.g., 10-m walk test (10mWT), timed 25-foot walk (T25FW), timed-up-and-go (TUG), 2-min walk test (2MWT)) in pwMS. Although supervised assessments performed in the clinic provide important information about improvement in functional capacity, they do not provide objective information about the impact of therapy in daily life (i.e., actual performance of the pwMS). The ecological validity of supervised clinical tests has been questioned, and discrepancies between mobility parameters measured in supervised and unsupervised settings have been demonstrated [14, 26, 57]. For example, the T25FW or 2MWT correlate only weakly with walking in free-living as reported in cross-sectional studies [17]. Unsupervised environments often involve challenging situations such as obstacles, busy corridors, or multitasking (e.g., walking and talking) that are not captured by standardized walking tests conducted in the laboratory.

Thus, in recent years, the field of home monitoring has gained interest, and several studies have examined physical activity (PA) in the daily lives of pwMS. The most commonly used outcome is steps count using actigraphy during several consecutive days [1, 14, 16, 17, 39, 58]. Two of these studies also assessed PA intensity using walking cadence [39] or metabolic equivalent of task [16]. These former studies yielded consistent results: pwMS tend to have a sedentary lifestyle with PA being significantly lower than the PA recommendations given by the World Health Organization. In addition, a significant negative correlation was found between the number of steps per day and the Expanded Disability Status Scale (EDSS) [1, 16]. Free-living actigraphy provides an opportunity to objectively assess the mobility of pwMS. However, the step/activity count approach only partially addresses the multidimensional aspect of PA. Recently, robust algorithms for ambulatory locomotion detection have been developed and validated that take into account different PA dimensions such as activity type, duration, and intensity [43, 48, 59]. These different PA dimensions can be combined to obtain a symbolic sequence of PA states, also called *“barcoding”* [44]. Remarkably, the symbolic time series of PA contain information about both the PA performed during the day (i.e., classical metrics) and the temporal fluctuation/organization of activities (i.e., complexity metrics). Previous studies have shown that the complexity of PA barcode significantly captures pain-related functional limitations in patients with chronic pain [44], concern about falling in older adults [42], or mobility limitations in young older adults [61].

In conjunction with daily PA, gait parameters are considered as important mobility-related measure [18, 54]. Among these metrics, gait speed is a dependable marker of functional decline [10, 47]. The main challenge in measuring free-living gait speed is the need for validated algorithms for reliable and accurate feature extraction. In recent years, researchers have developed robust algorithms for locomotion detection in a free-living environment using trunk-mounted (chest/lower back) [30, 43, 59], or foot-mounted inertial measurement unit (IMU) [48]. The advantage of the foot-mounted IMU is the clinically acceptable accuracy of the gait spatio-temporal parameters which can be further extracted [31]. The distribution of walking speed in daily life has already been studied in patients with Parkinson's disease and showed promising results [4]. However, to the best of our knowledge, no previous work has investigated gait parameters in free-living environment in pwMS, and the effects of a rehabilitation period on PA and gait.

Therefore, our project’s primary objective is to bring new insights into the impact of a multidisciplinary inpatient rehabilitation (MIR) on self-reported questionnaires, supervised walking tests, PA behavior and everyday gait patterns using shoe-attached IMUs. We also consider the disability level, distinguishing between mildly and severely disabled pwMS. Then, our secondary objective is to compare and discuss gait speed measured in free-living conditions (i.e. unsupervised) with gait speed measured in the clinic (i.e. supervised), as their difference is an important outcome for the evaluation of an intervention [4, 57]. Supervised assessments performed in the clinic reflect functional capacity, whereas unsupervised assessments during daily activities are indicative of the actual performance of the patients [20].

## Methods

### Participants and study design

A total of 52 patients diagnosed with MS (EDSS: 4—6.5; age ≥ 18 years) were included in the study. PwMS with severe cognitive or arm/hand impairments interfering with study participation, or with comorbidities, such as musculoskeletal or cardiovascular diseases, that reduced walking ability were excluded from the study. In addition, pwMS with EDSS scores greater than 6.5 were excluded. The study was approved by the Ethical Committee of eastern Switzerland (EKOS, 2017-00728), and performed in agreement with the Declaration of Helsinki’s Ethical Principles for Medical Research Involving Human Subjects. For provisional inclusion, pwMS who were registered for planned rehabilitation were contacted by telephone by a researcher who verified the inclusion criteria and provided verbal information about the study. After provisional inclusion, a letter was sent to patients with written information about the study, an informed consent form, a box containing two IMUs (Physilog® 5, Gait Up, CH) with instructions, and a set of questionnaires. Definite inclusion was at the start of rehabilitation, when inclusion criteria and patients’ ability to use the sensors were checked.

**Fig. 1:**
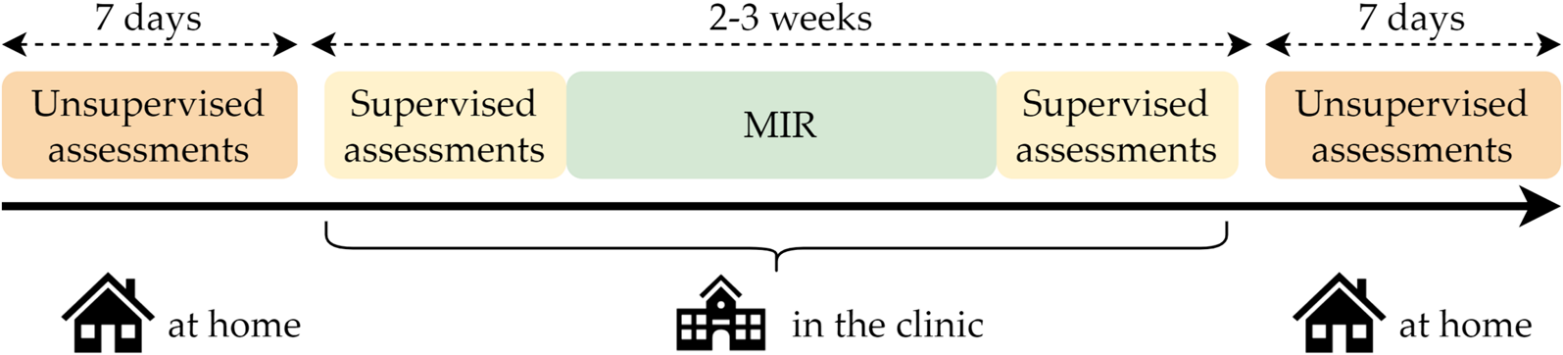
Study design overview. Measurement pre-rehabilitation: 7 consecutive days within 1–4 weeks before the multidisciplinary inpatient rehabilitation (MIR), depending on patients’ availabilities; measurement post-rehabilitation: 7 consecutive days immediately after the MIR

PwMS were evaluated pre- and post- MIR as explained in Fig. 1. Each measurement period consisted of clinical assessments (i.e. supervised), and 7 days home monitoring (i.e. unsupervised). The personalized MIR program, of 2 to 3 weeks, generally included the following components: balance and walking training (physiotherapy, 5 times/week), strength training (3 times/week), aerobic exercise training (3 times/week), occupational therapy focusing on energy management and activities of daily living (2–3 times/week) [19], and neuropsychological training (2 times/week). The program was adapted to every patient to ensure an appropriate training in terms of difficulty (e.g. free overground walking vs walking with weight support, flat vs uneven ground, difficult (steep) vs easy (wide, not steep, with handrails) stairs). There were no specific interventions to target daily levels of PA.

#### Clinical assessments and supervised tests

Each clinical assessment included a set of questionnaires: Fatigue Scale for Motor and Cognitive Functions (FSMC), patient-reported walking ability (Twelve Item Multiple Sclerosis Walking Scale (MSWS-12)), and the patient's self-rated health on a vertical visual analogue scale (EQ-VAS, a sub-score of the EQ-5D-5L questionnaire). Three scores were reported for the FSMC questionnaire: the total score (FSMCt), the motor sub-score (FSMCm) and the cognitive sub-score (FSMCk). Then, pwMS were asked to perform three walking tests to objectively measure their functional capacity: the timed-up and go (TUG), the 10 m walk test (10mWT) at fast speed, and the 2 min walk test (2MWT). The time required to perform the TUG and 10mWT was measured with a stopwatch. The Expanded Disability Status Scale (EDSS) was evaluated by a neurologist.

#### Home assessments

PwMS were asked to wear two sensors (Physilog® 5, Gait Up, CH) for seven days, one fixed on each foot (shoe), after getting dressed in the morning and to take it off before going to bed. Participants were allowed to go outside the home and perform their usual activities. Therefore, home assessments can also include daily activities that had been done outside their living space. The sensors were programmed to start recording automatically at 9:00 for 12 h, i.e., until 21:00. To promote adherence to the protocol, physiotherapists contacted each pwMS during the home measurement period. This was done to ensure they understood the protocol clearly and to address any potential issues related to sensor usage. In addition, participants were offered the choice to receive morning reminders for wearing the sensors and evening reminders for charging them. A day of measurement was considered valid if the sensors were worn for, at least, 8 h. Participants with three or more valid days per period (i.e., pre- and post- rehabilitation) were included in the analysis. A minimum of three days is necessary for a reliable estimation of usual behaviour [1, 38]. In order to have the same data length for all participants and all days, the data were segmented to obtain exactly 8 h of wearing time per day, starting from the first movement detected.

### Feature extraction from home assessments

#### Locomotion detection

The locomotion periods or walking bouts (WBs) were automatically extracted using a previously validated algorithm [48]. The algorithm is based on a peak enhancement filtering method using continuous wavelet transforms of the triaxial angular velocity norm recorded at the foot. Only the walking episodes that contained at least two consecutive strides (i.e. 5 steps) are considered as true locomotion [24]. The accuracy of locomotion detection is crucial for the correct calculation of gait and PA metrics.

#### Gait metrics

With a previously validated gait analysis algorithm [31], the gait parameters were extracted for each stride within each WB. In order to have more steady-state gait, very short WBs with less than 6 detected strides per foot were removed. Then, for each gait cycle, the speed, cadence, stride length and gait cycle time were computed. Finally, for each WB, we calculated the mean value of the gait parameters extracted per gait cycle. There is no clear consensus on the granularity by which the gait parameters should be assessed (i.e., stride-wise, averaged over WBs or averaged over time intervals based on multiple WBs) [24]. However, it seems well-accepted to compute the walking speed over a minimal number of consecutive strides, which agrees with the definition of a WB. We thus decided to adopt the WB granularity to extract the digital mobility outcomes as done in previous studies [57].

#### Physical activity (PA) metrics

Based on the type of activity (locomotion, non-locomotion), duration (very short, short, medium, long) and intensity (acceleration magnitude, cadence), PA was divided into 25 states [42], starting from lowest state (state 1) to highest states (state 25), also called "barcode" (details are provided in appendix 1 in supplementary material). Three classical metrics were evaluated; percentage of daily locomotion (Loc), light PA (LPA) and moderate-to-vigorous PA (MVPA) per day. A range from 83 to 104 steps/min, depending on the subject’s height, was found to correspond to 3 metabolic equivalent of task (METs), which is commonly used as the threshold between light to moderate exercise intensities [3]. Consequently, we choose 90 steps/min as an appropriate cut-off between LPA and MVPA. The number of steps per day, and the WB duration were also computed. Then, two complexity metrics were calculated to characterize the temporal fluctuations of the daily PA patterns; the information entropy (Hn) and the permutation Lempel–Ziv complexity (PLZC). The Hn is defined as a structural-static complexity metric which characterizes the amount of different states in the barcode. If there are many (few) different types of PA states in the barcode, Hn takes a large (small) value. Hn is sensitive to the variety of PA states, however is insensitive to the temporal ordering of the sequence. PLZC is a structural-dynamic metric that quantify the temporal behaviour (i.e. ordering of different states). This metric captures the number of distinct sub-strings and their rate of occurrence as the sequence evolves from left to right [7].

### Data aggregation

#### PA metrics in daily life

The classical and complexity metrics were extracted per day from the barcode. Consequently, we expect between 3 and 7 values per subject and per measurement period depending on the number of valid days analysed. In such study design, in which multiple observations from the same participant are collected, the linear mixed-effect (LME) model is particularly well-adapted [55]. This statistical model considers the repeated measures data nested within-subject as the level-1 of the linear regression. As a consequence, the multiple values obtained from the same patient did not need to be further aggregated by using the mean or median for example.

#### Gait pattern in daily life

**Fig. 2:**
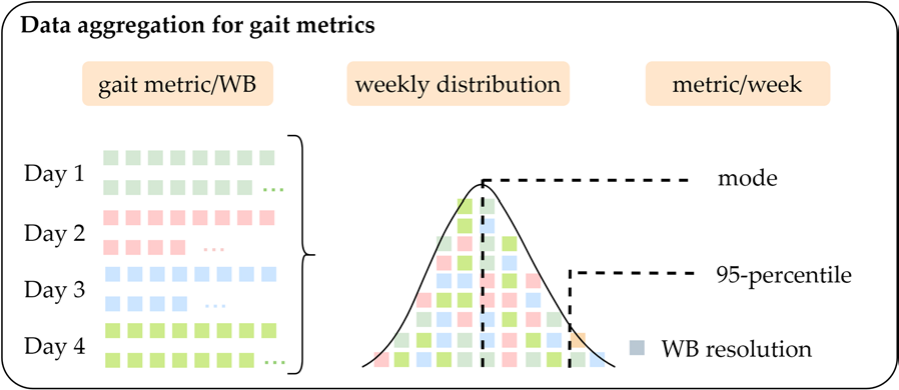
Data aggregation procedure for the gait metrics assessed in daily life. *WB:* walking bout

The gait metrics were computed per WB (i.e., WB granularity). In terms of gait assessment, contrarily to the PA metrics, we were not interested in a daily-based behavior, rather the general behavior at home. Consequently, the gait parameters from all the measurement days were aggregated to obtain a unique weekly distribution (as illustrated in Fig. 2). We then computed the kernel density smoothening function, a non-parametric method to estimate the probability distribution function (pdf), to extract the mode and the 95th-percentile, giving a statistically robust representation of the patient’s gait performances in daily living. The mode (i.e., the value that occurs most frequently in a set of data values) represents the patient’s usual gait pattern, and the 95th-percentile, on the other hand, refers to the maximum performance that patients perform in daily life. We reported the results of a subset of the extracted metrics based on their meaningful clinical interpretations. We thought it relevant to report the mode of cadence, gait speed, and stride length as the patient's usual gait pattern. In addition, we included the 95th-percentile of the WB time, gait speed, and the stride length, because we expect these parameters to capture endurance and gait capacity improvements.

### Statistical analysis

A linear mixed-effects model (*LME*) was applied to investigate the influence of the rehabilitation (i.e., pre vs. post-intervention) on the clinical assessments (i.e., questionnaires and functional tests) and home measurements (i.e., PA and gait metrics). This statistical analysis method allows to test relations among both within- and between-levels data without violating standard assumptions of independence. In addition, *LME* accommodates missing data. We considered two subgroups, *‘mild’* (EDSS < 5) and *‘severe’* (EDSS ≥ 5) disability levels based on the EDSS scores. A 2-levels *LME* model was designed with the *“rehab”* (i.e., ‘pre’ vs. ‘post’), the *“group”* (i.e., ‘mild’ vs. ‘severe’), and the interaction between *“rehab”* and *“group”* as the fixed effects (see **Eq. **1). Then, a random effect (intercept and slope) at the subject level was defined to consider the repeated measures data nested within-subject (i.e., *“(rehab|subject)”* in **Eq. **1). The following equation was used as input to the “*fitlme”* MATLAB function (with the *“responder”* corresponding to PA or gait metric):

1 $${\text{responder}}\hspace{0.17em}\sim \hspace{0.17em}\mathrm{rehab }*\mathrm{ group}\hspace{0.17em}+\hspace{0.17em}({\text{rehab}}|{\text{subject}})$$

This model corresponds to.

$$\mathrm{Level }1:{y}_{ij }= {\beta }_{0j}+ {\beta }_{1j}({rehab)}_{ij} + {\varepsilon }_{ij}$$

2 $$\mathrm{Level }2:{\beta }_{0j}= {\gamma }_{00} + {\gamma }_{01}{(group)}_{j}+ {\mu }_{0j}; {\beta }_{1j}= {\gamma }_{10} + {\gamma }_{11}{(group)}_{j} + {\mu }_{1j}$$

The overall model:

3 $${y}_{ij }= {\gamma }_{00} + {\gamma }_{01} ({group)}_{j}+ {\gamma }_{10} ({rehab)}_{ij}+ {\gamma }_{11}{ \left(rehab\right)}_{ij}*{\left(group\right)}_{j}+ {\mu }_{0j}+ {\mu }_{1j} ({rehab)}_{ij}+ {\varepsilon }_{ij}$$

where $i=1, 2,\dots , n,$ (*n* is the number of observations), $j=1, 2,\dots , 43,$ corresponds to the pwMS. The model estimates (${\gamma }_{00}$, ${\gamma }_{01}$, ${\gamma }_{10}$, and ${\gamma }_{11}$), p-value, and 95% confidence interval (CI) values of the fixed effects were used to understand apparent significant effects. Statistical significance was accepted if p ≤ 0.05 and if the lower and upper limits of the 95% CI did not include 0. In addition, the conditional ${R}_{c}^{2}$ was computed to assess the total variance explained by both fixed and random effects.

## Results

### Participants

Of the 52 pwMS involved in the study, 4 were not assessed after the rehabilitation due to unavailability for personal reasons. The therapy was provided as part of an inpatient program at the clinic, where the adherence was closely monitored. Missed sessions were usually rescheduled later in the same week. Across all pwMS, irrespective of their level of disability, the average total weekly rehabilitation duration was about 790 min. From this group, 43 pwMS met the inclusion criteria of, at least, three valid days per measurement period. The average number of valid days recorded at pre- and post- intervention were 5.5 ± 1.9 and 6.4 ± 1.2 days respectively. In total 631 valid days were analyzed. The average start and stop time were 09:06 ± 00:20 a.m. and 05:06 ± 00:20 p.m. respectively. The Table 1 provides the characteristics of the pwMS involved in the rehabilitation program.

**Table 1:** Clinical characteristics of the MS study group

Variable	Entire group (n = 43)	Mild disability (EDSS < 5) (n = 19)	Severe disability (EDSS > = 5) (n = 24)
Average age (mean ± std) (years)	51.7 ± 11.0	47.7 ± 9.9	54.8 ± 11.0
Age (min; max) (years)	28; 75	28; 63	34; 75
Female (n; %)	26; 60.5	10; 52.6	16; 66.7
Male (n; %)	17; 39.5	9; 47.4	8; 33.3
Disease duration (mean ± std) (years)	13.2 ± 9.7	9.7 ± 7.9	16 ± 10.2
Average EDSS (mean ± std)	5.2 ± 1.4	3.1 ± 0.8	5.9 ± 0.6
EDSS (min; max)	2; 6.5	2; 4	5; 6.5

### Effects of MIR on self-reported questionnaires

**Fig. 3:**
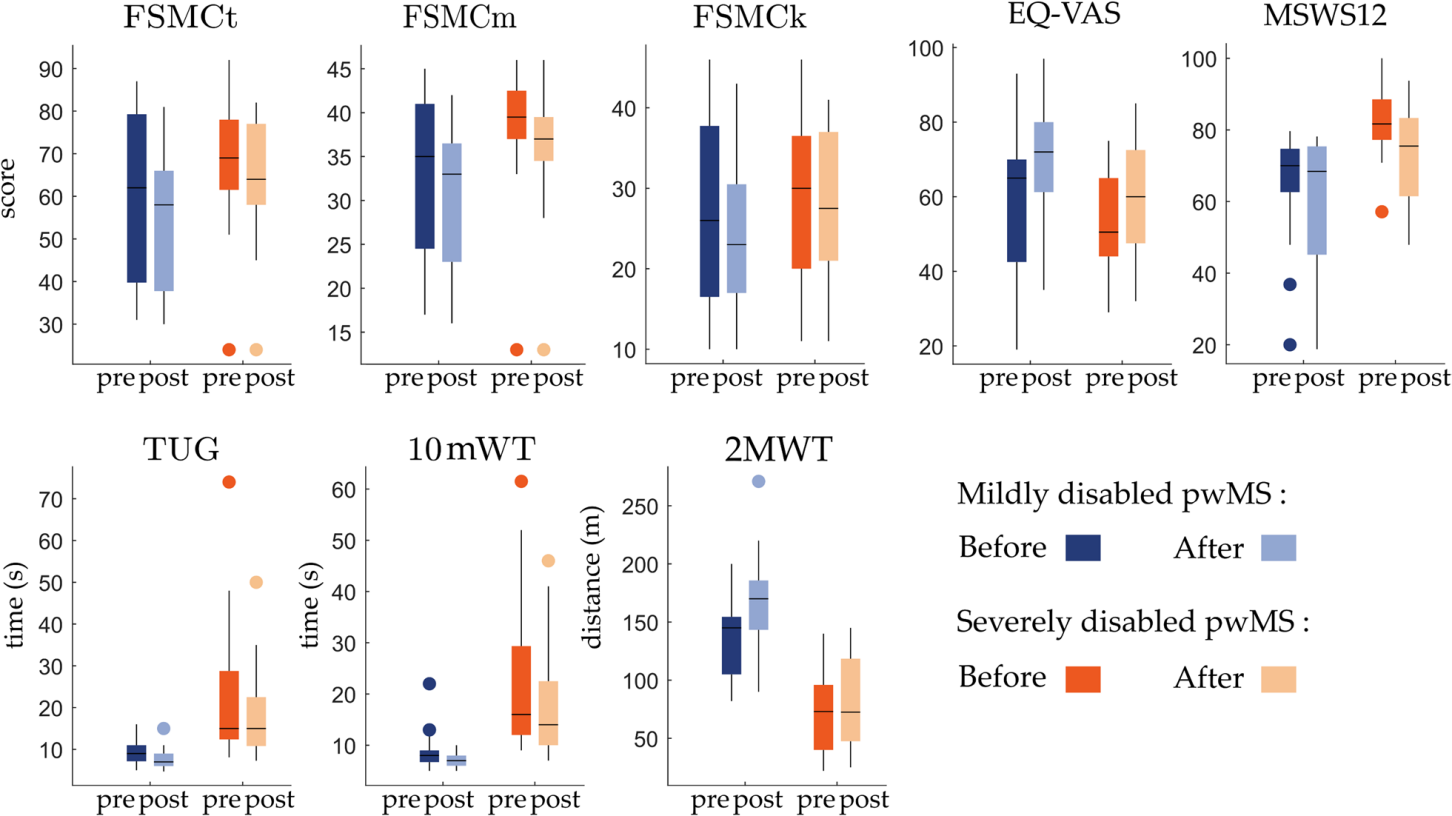
Patient-reported questionnaires and functional tests pre- and post- rehabilitation. The boxplots on the left (dark and light blue) correspond to the scores obtained for mildly disabled pwMS, whereas the boxplots on the right (dark and light orange) summarize the values obtained for severely disabled pwMS. *FSMC* Fatigue Scale for Motor and Cognitive Functions, *EQ-VAS* patient’s self-rated health on a vertical visual analogue scale, *MSWS-12* Twelve Item Multiple Sclerosis Walking Scale, *TUG* timed-up and go, *10mWT* 10 m walking test, *2MWT* 2 min walking test, *EDSS* Expanded Disability Status Scale

The scores of the five self-reported questionnaires (i.e., FSMCt, FSMCm, FSMCk, EQ-VAS, MSWS-12) assessed before and after rehabilitation are shown in Fig. 3. The statistical analyses based on the *LME* models (appendix 2 in supplementary material**)** indicate significant effects of the rehabilitation period on the FSMCt, FSMCm, EQ-VAS, and MSWS-12 questionnaires (p ≤ 0.05 for ${\gamma }_{10}$-estimate, appendix 2 in supplementary material). Interestingly, we observe significant differences between groups for the FCMSm and MSWS-12 questionnaires (p ≤ 0.05 for ${\gamma }_{01}$-estimate, appendix 2 in supplementary material), both of which evaluate motor capacity functions.

### Effects of MIR on supervised walking tests

The results obtained for the supervised walking tests (i.e., TUG, 10mWT, and 2MWT) indicate significant effects for both the *“group”* and *“rehab”* estimates (Fig. 3 and table in appendix 2 in supplementary material), meaning a significant difference between mildly and severely disabled pwMS and an improvement after the intervention. Furthermore, the significant interaction effect “group*rehab” (p ≤ 0.05 for ${\gamma }_{11}$-estimate, appendix 2 in supplementary material) for the 2MWT highlights that one group improved more than the other. Figure 3 illustrates this trend clearly, the mildly disabled pwMS experienced a higher increase in the walking distance than those with severe disabilities.

### Effects of MIR on physical activity and gait in daily life

#### Effects of MIR on PA behavior

**Fig. 4:**
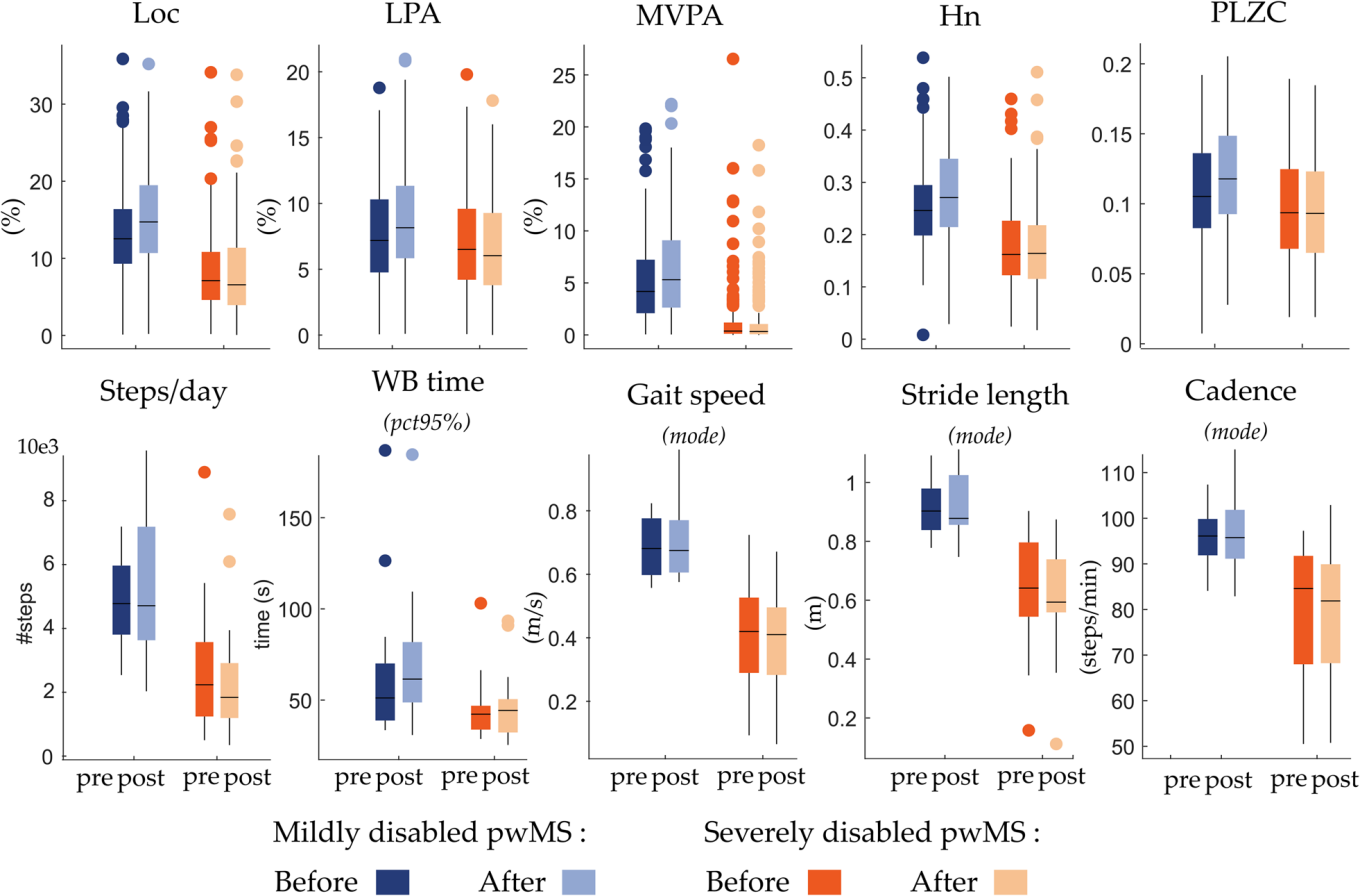
Physical activity (PA) and gait metrics pre- and post- rehabilitation. *Loc* percentage locomotion per day, *LPA* percentage of light PA per day, *MVPA* percentage of moderate-to-vigorous PA per day, *Hn* information entropy, *PLZC* permutation Lempel–Ziv complexity;

As can be seen in Table 2 and Fig. 4, there are significant interaction effects (i.e., “group*rehab”, p ≤ 0.05 for ${\gamma }_{11}$-estimate) for *Loc* and the two complexity metrics; Hn and PLZC. Mildly disabled pwMS significantly increased their percentage of locomotion per day, and complexity of daily PA pattern. Increasing, but not significant, trends of the remaining PA metrics (i.e. LPA, MVPA, and #steps/day) are observed for the mildly disabled pwMS (“group*rehab”, ${\gamma }_{11}$-estimate in Table 2). In addition, the Loc, MVPA, #steps/day and Hn metrics are significantly higher for the mildly disabled than the severely disabled pwMS (p ≤ 0.05 for ${\gamma }_{01}$-estimate, Table 2) regardless of rehabilitation (i.e., pre- vs. post-rehabilitation).

**Table 2:** LME models obtained for PA and gait metrics in daily life assessments

Responder	Predictor	$\gamma$	Estimate	|t|	p	95% CI for estimate		Total predict (${R}^{2}$)
Loc (%)	Intercept	${\gamma }_{00}$	8.38					0.64
	Rehab	${\gamma }_{10}$	− 0.38	− 0.68	0.497	− 1.50	0.73	
	Group	${\gamma }_{01}$	**5.10**	**3.924**	0.000*	**2.55**	**7.65**	
	Group*rehab	${\gamma }_{11}$	**1.98**	**2.303**	0.022*	**0.29**	**3.66**	
LPA (%)	Intercept	${\gamma }_{00}$	7.05					$0.49$
	Rehab	${\gamma }_{10}$	− 0.30	− 0.79	0.427	− 1.03	0.44	
	Group	${\gamma }_{01}$	1.02	1.149	0.251	− 0.72	2.77	
	Group*rehab	${\gamma }_{11}$	1.03	1.810	0.071	− 0.09	2.14	
MVPA (%)	Intercept	${\gamma }_{00}$	1.33					$0.75$
	Rehab	${\gamma }_{10}$	− 0.08	− 0.23	0.815	− 0.76	0.60	
	Group	${\gamma }_{01}$	**4.10**	**4.597**	0.000*	**2.35**	**5.85**	
	Group*rehab	${\gamma }_{11}$	0.92	1.759	0.079	− 0.11	1.96	
Hn	Intercept	${\gamma }_{00}$	0.18					$0.62$
	Rehab	${\gamma }_{10}$	0.00	− 0.65	0.515	− 0.02	0.01	
	Group	${\gamma }_{01}$	**0.08**	**4.321**	0.000*	**0.04**	**0.11**	
	Group*rehab	${\gamma }_{11}$	**0.03**	**2.500**	0.013*	**0.01**	**0.05**	
PLZC	Intercept	${\gamma }_{00}$	0.10					$0.50$
	Rehab	${\gamma }_{10}$	0.00	− 1.46	0.146	− 0.01	0.00	
	Group	${\gamma }_{01}$	0.01	1.614	0.107	0.00	0.03	
	Group*rehab	${\gamma }_{11}$	**0.01**	**2.653**	0.008*	**0.00**	**0.02**	
#steps/day	Intercept	${\gamma }_{00}$	2584.85					$0.92$
	Rehab	${\gamma }_{10}$	− 284.55	− 1.03	0.305	− 833.30	264.20	
	Group	${\gamma }_{01}$	**2186.91**	**4.28**	0.000*	**1169.65**	**3204.16**	
	Group*rehab	${\gamma }_{11}$	723.66	1.77	0.081	− 92.22	1539.54	
WB time (s) pct95%	Intercept	${\gamma }_{00}$	44.43					$0.69$
	Rehab	${\gamma }_{10}$	1.54	0.26	0.795	− 10.23	13.32	
	Group	${\gamma }_{01}$	**18.80**	**2.21**	0.030*	**1.87**	**35.72**	
	Group*rehab	${\gamma }_{11}$	4.01	0.46	0.650	− 13.49	21.51	
Cadence (steps/min) mode	Intercept	${\gamma }_{00}$	80.06					$0.98$
	Rehab	${\gamma }_{10}$	− 0.48	− 0.52	0.605	− 2.30	1.35	
	Group	${\gamma }_{01}$	**15.72**	**4.60**	0.000*	**8.92**	**22.53**	
	Group*rehab	${\gamma }_{11}$	1.37	1.00	0.319	− 1.35	4.09	
Gait speed (m/s) mode	Intercept	${\gamma }_{00}$	0.41					$0.97$
	Rehab	${\gamma }_{10}$	− 0.01	− 0.45	0.654	− 0.03	0.02	
	Group	${\gamma }_{01}$	**0.28**	**7.02**	0.000*	**0.20**	**0.36**	
	Group*rehab	${\gamma }_{11}$	0.02	1.00	0.322	− 0.02	0.06	
Gait speed (m/s) pct95%	Intercept	${\gamma }_{00}$	0.62					$0.99$
	Rehab	${\gamma }_{10}$	0.00	0.01	0.993	− 0.03	0.03	
	Group	${\gamma }_{01}$	**0.45**	**8.99**	0.000*	**0.35**	**0.55**	
	Group*rehab	${\gamma }_{11}$	0.03	1.43	0.156	− 0.01	0.08	
Stride length (m) mode	Intercept	${\gamma }_{00}$	0.63					$0.97$
	Rehab	${\gamma }_{10}$	− 0.02	− 1.45	0.152	− 0.05	0.01	
	Group	${\gamma }_{01}$	**0.28**	**6.38**	0.000*	**0.19**	**0.37**	
	Group*rehab	${\gamma }_{11}$	0.03	1.43	0.156	− 0.01	0.07	
Stride length (m) pct95%	Intercept	${\gamma }_{00}$	0.87					$0.97$
	Rehab	${\gamma }_{10}$	**0.03**	**2.09**	0.040*	**0.00**	**0.07**	
	Group	${\gamma }_{01}$	**0.36**	**7.93**	0.000*	**0.27**	**0.46**	
	Group*rehab	${\gamma }_{11}$	− 0.01	− 0.27	0.785	− 0.06	0.04	

Bold values with an asterisk (*) correspond to significant results, indicated by p-value < 0.05 and 95% confidence interval (CI) that do not include 0

#### Effects of MIR on gait pattern in daily life

**Fig. 5:**
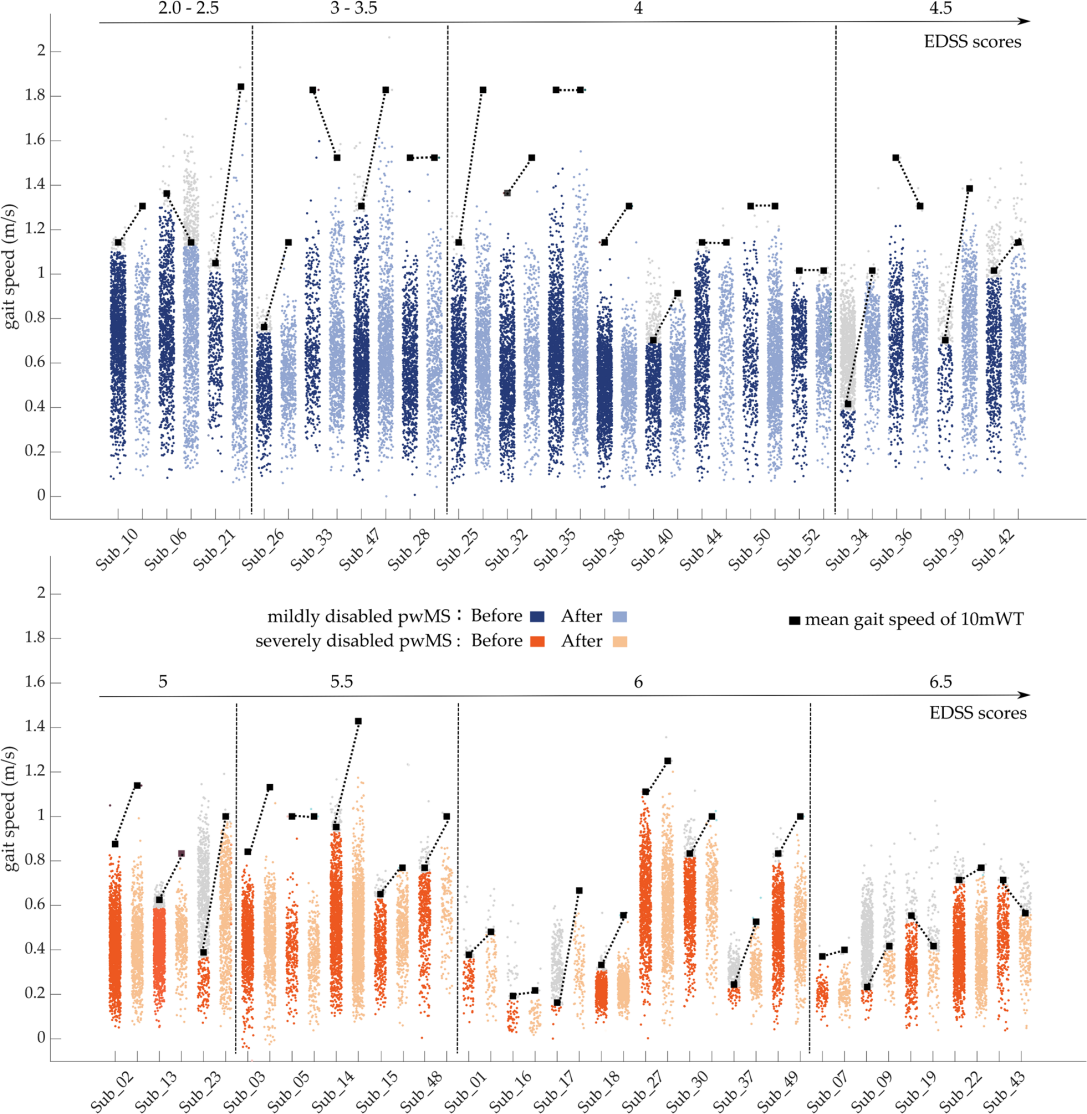
Distribution of gait speed at home (small color dot) for each patient before and after the rehabilitation period. Each dot corresponds to the average walking speed during one walking bout. The large black squares represent the average values of the 10 m walking test assessed in supervised condition

What stands out in Fig. 4 and Table 2 are the clear and significant differences between mildly and severely disabled pwMS (i.e., *“group”* effects, ${\gamma }_{01}$-estimates, Table 2) for all reported gait metrics. However, our LME models do not show significant *“rehab”* effect for all reported gait metrics. The gait speed distributions at home and the average values of the gait speed measured in supervised conditions (10mWT) for each patient before and after the rehabilitation period are provided in the Fig. 5. First of all, we notice that the average walking speed obtained during the 10mWT lay at the extreme end of the gait speed distribution measured at home, and increase post-rehabilitation. Another striking observation to emerge from Fig. 5 is the decrease in gait speed as the EDSS scores increase.

## Discussion

### Effect of MIR on self-reported questionnaires and supervised walking tests

#### Self-reported questionnaires

In the present MS cohort, mildly and severely disabled pwMS reported significant improvements in their walking ability as assessed by the MSWS-12 questionnaire (-6.6 and -9.4 points, respectively), which is in the range of clinically meaningful changes between -6 and -11 points [5, 6]. The improvement in fatigue (FSMC) by -4 and -3 for mildly and severely disabled pwMS, respectively, is consistent with previous studies [26, 33], but below the reported minimum for a clinical meaningful change (-9 points) [53]. In addition, pwMS also reported significant improvements in their self-rated health (EQ-VAS) of about + 10 and + 9 points for mildly and severely disabled pwMS (see appendix 3 in supplementary material).

#### Supervised walking tests

The significant improvements in walking capacity during the 2MWT, measured in both mildly (mean ± std: + 30.2 ± 36.0 m) and severely (mean ± std: + 11.7 ± 16.8 m) disabled pwMS, exceed the reported minimum for clinically relevant changes (i.e., 9.6 m) [5]. Similarly, the 10mWT improvements reach approximately -13.9% and -20.0% for mildly and severely disabled pwMS, nearing the clinically relevant change of -23% [40]. Then, TUG test was assessed as a complementary measure of functional mobility for activities such as sitting, standing or turning around [49]. The improvements measured in TUG test of 15.4% and 12.0% in the mildly and severely disabled pwMS, respectively, are statistically significant but lower than the established threshold of 23% for genuine changes [40]. Our results confirm previous findings demonstrating statistically significant improvements in all clinical walking outcome measurements (i.e., 10mWT, 2MWT and TUG) after a physical rehabilitation program [13, 14, 22, 26]. Taken together, these results demonstrate that pwMS with mild and severe disabilities benefit from the MIR by significantly and clinically improving their perceived and actual functional mobility.

### Effects of MIR on physical activity and gait in daily life

#### Physical activity in daily life

Consistent with the literature, pwMS with mild walking disability do more steps per day (~ 5000 steps/day, Table 2) than pwMS with severe disability (~ 2500 steps/day, Table 2) [14, 39]. Daily step count reliably reflects unsupervised daily-life walking behavior in pwMS. An increase of about 800 steps/day indicates clinically meaningful progress following an intervention [35]. While mildly disabled pwMS showed a rising trend, our cohort did not exhibit this enhancement. Then, a minor but significant increase in daily percentage locomotion is noted in mildly disabled pwMS. The intensity and duration of locomotion are also a critical aspect to consider as being related to cardiovascular health. Our results demonstrate that pwMS in the mildly disabled subgroup clearly walk more minutes at moderate intensity (mean ± std: 5.0 ± 2.9%) than pwMS with severe MS (mean ± std: 1.3 ± 2.8%, appendix 4 in supplementary material). Then, the maximum WB duration (i.e., 95th-percentile of the long tail distribution) provides information about pwMS’ ability to walk continuously for a certain time. Again, our findings highlight that mildly disabled pwMS (EDSS < 5) walk longer than severely disabled pwMS (Table 2, and Fig. 4). Consistent with prior studies, pwMS in our cohort rarely engage in uninterrupted 2-min walks, and even struggle with 1-min walks for those with severe MS disabilities (Table 2, and Fig. 4), and predominantly at low intensity [39]. Despite the daily locomotion percentage, the statistical LME models do not uncover significant rehabilitation impact on conventional PA metrics.

In addition to the above conventional PA metrics, we investigated the complexity of daily PA. Notably, the information entropy (Hn) is significantly higher for mildly disabled than severely disabled pwMS. Interestingly, the complexity of the daily PA time-series, assessed by Hn and PLZC, of mildly disabled pwMS, significantly increase after the MIR. These results may be explained by additional PA states in barcodes, most likely tied to longer WBs or higher cadence.

#### Gait patterns in daily life

All of the reported gait parameters (i.e., gait speed, cadence, and stride length) demonstrate highly significant differences between the two subgroups of pwMS (Fig. 4). These results confirm the evidence of using gait parameters, particularly gait speed, as discriminative features for disease severity. The usual cadence (mild disability: 95.8 ± 6.2 steps/min; severe disability: 80.1 ± 14.2 steps/min) and gait speed (mild disability: 0.7 ± 0.09 m/s; severe disability: 0.4 ± 0.16 m/s) measured in our cohort are notably lower than the reference values reported for healthy subjects on daily living (cadence: 118.86 ± 6.76 steps/min; gait speed: 1.3 ± 0.1 m/s) [41]. The 95th-percentile of the weekly speed distribution informs about an individual’s maximal performance. Our results indicate that mildly disabled pwMS are able to reach gait speed values of approximately 1.1 m/s, close to the preferred walking speed of healthy subjects, in specific free-living conditions if needed. However, severely disabled pwMS could not walk faster than about 0.6 m/s (appendix 4 in supplementary material), which might limit their participation in activities of daily living (ADLs). In the pre-post comparison, our findings indicate that while patients enhance their capacity significantly, their walking performance in free-living environment does not show significantly improvements (Table 2, Fig. 4).

#### Capacity vs. performance

The Fig. 5 nicely highlights important aspects of our current study, and mainly points out that pwMS do not make a direct use of their capacity improvements into their activities in daily life. Indeed, 37 out of 40 pwMS walked faster or longer during the 2MWT and 10mWT, while they did not change their walking performance in daily life. The capacity and performance measures should be considered as complementary assessments. The 2MWT, 10mWT and TUG are meaningful clinical measure and well-established tests to assess walking function [9]. In the majority of our results, the walking tests with fast speed lay at the extreme end of the gait speed distribution at home (Fig. 5), which is in line with what has been previously reported in the literature [4, 56, 57]. Previous studies already argue that supervised walking tests are a “snapshot” of walking functions measured at a specific time and on a specific day, and assess only part of the variance in daily step count. Indeed, other factors (i.e., fluctuating symptoms, such as fatigue, mood, spasticity or daily form/condition, or environmental aspects) not necessarily captured by the supervised walking tests, might highly influence daily activities [23, 52]. Unsupervised assessments conducted over several days include the best and worst behaviors in relation to the above-mentioned influencing factors.

Notably, mildly disabled pwMS are slightly more active in their daily lives after the rehabilitation period. It seems that these pwMS do not use their improved abilities to walk faster, but to walk more (i.e., increased locomotion, Hn, and LPZC). This finding is quite intuitive, since in everyday life people would rather walk at their preferred speed than exert themselves without a specific goal. These capacity improvements can be interpreted as a higher *"physiological reserve"* that could help pwMS to engage more in ADLs, and might also explain the higher reported self-rated health (EQ-VAS). With regards to the severely disabled pwMS, their overall walking capacity improved, but neither PA behavior nor gait pattern in daily life changed. PwMS who walk at slow speeds, both in supervised and unsupervised conditions, may need to challenge themselves in ADLs. In a survey assessing the impact of walking speed on ADLs, a majority of pwMS reported walking impairments as the most challenging aspect of their disease [60]. Particularly, slow walking speed might limit MS patients to execute basic activities such as crossing the street, or walking to the nearest shop. The ability to speed up over a short distance is of high concern for severely disabled pwMS, and leads sometimes to ADLs avoidance [60]. Despite an improvement in walking capacity, certain environmental barriers (e.g., ascents/descents, stairs, etc.) might still pose unsurmountable challenges for severely disabled pwMS.

## Limitations

Home assessment on multiple measurement days is a very challenging protocol, and several limitations must be acknowledged. First, for practical reasons, we asked patients to equip themselves with the IMUs each morning. Despite our efforts to properly instruct pwMS during the supervised test sessions and well-documented paper guidelines, we do not have a complete guarantee that the sensors were properly attached. It should be noted that the orientation of the sensors on the shoes is not crucial, as our algorithms are designed to be robust to different placements of the sensors [31, 32]. However, if the rubber clip is not properly attached to the laces, interfering vibrations of the sensor at heel strike can affect the signal. Despite our data quality checking procedure, we may have missed some noisy signals. Considering the large amount of data collected (more than 600 files), we believe that a few errors in the gait parameters at the stride level do not affect the overall weekly distribution and the associated mode and 95th percentile. Then, despite the comprehensive database, additional participants would have allowed stratification into three subgroups (i.e., EDSS 2–3; EDSS 3.5–4.5; and EDSS > 5). Differentiating between pwMS with low disability (EDSS < 3) and pwMS with moderate disability (EDSS: 3.5–4.5) could have provided more insight into the impact of MIR on these subgroups. Finally, other potential confounding factors (education, employment status, comorbidity, season/weather, occupation, medical leave, usage of walking aids, etc.) were not available to be included in the statistical models.

## Practical implications

Exercise training has been proven to be effective in improving leg muscle strength, balance, cardio-pulmonary fitness, and walking capacity in pwMS [28, 45, 51]. This statement is in line with our findings showing significant improvements in walking capacity (as assessed by 2MWT, 10mWT, and TUG) and self-reported questionnaires (i.e., MSWS-12, FSMC, EQ-VAS) after rehabilitation. However, those walking capacity improvements do not necessarily translate into increased mobility in daily life, especially for severely disabled pwMS. Supervised clinical assessments help diagnose patients, set goals, and prescribe PA interventions. However, practitioners should not only focus on walking capacity, but also consider behavioral and psychological (e.g., motivation, or self-efficacy) factors that might influence PA behavior and walking performance in the long term [11, 36, 37]. Following inpatient rehabilitation, a personalized program [2, 21, 29], including achievable goals, barriers management strategies, motivation and self-efficacy enhancement, might be promising to motivate pwMS to increase their walking performance, such as spending more time walking, more time in moderate to vigorous PA or undertaking longer walking bouts in daily life. As evidenced in our study, mildly disabled pwMS slightly increased their PA following rehabilitation, a trend that is not observed among their severely disabled counterparts. Therefore, educational, motivational, and PA support strategies should begin early after diagnosis [15, 39]. Finally, wearable sensors could be used not only for research purposes but also as motivational tools for patients. Feedback can be implemented to help patients become aware of their daily PA and achieve their goals.

## Conclusion

First of all, our study demonstrates that mildly disabled pwMS walk more (i.e., locomotion and #steps/day), faster (i.e., gait speed), and longer (i.e., WB duration) in daily life than severely disabled pwMS. Secondly, our study provides comprehensive evidence of the beneficial effects of a MIR on self-reported mobility, self-rated health questionnaires, and walking capacity measured with supervised walking tests in both subgroups (i.e., mildly and severely disabled pwMS). However, these improvements do not necessarily translate to home performance in severely disabled pwMS, or only marginally mildly disabled pwMS. Motivational and behavioral factors should also be considered and incorporated into treatment strategies. As a follow-up to the MIR, future studies should examine the long-term effects of a personalized home or partly home-inpatient program on mobility, PA behavior, motivation, and well-being.

### Supplementary Information

Supplementary Material 1.

## Data Availability

The datasets generated and/or analyzed during the current study are not publicly available due to a lack of patient consent but are available from the corresponding author on reasonable request.

## Abbreviations

10mWT: 10-Meter walk test
2MWT: 2-Minute walk test
ADLs: Activities of daily living
EQ-VAS: Patient's self-rated health on a vertical visual analogue scale
FSMC: Fatigue Scale for Motor and Cognitive Functions
Hn: Information entropy
IMU: Inertial measurement unit
LME: Linear mixed-effect
LPA: Light physical activity
MIR: Multidisciplinary inpatient rehabilitation
MS: Multiple sclerosis
MSWS-12: Twelve Item Multiple Sclerosis Walking Scale
MVPA: Moderate-to-vigorous physical activity
PA: Physical activity
PLZC: Permutation Lempel–Ziv complexity
pwMS: People with multiple sclerosis
TUG: Timed-up-and-go
WB: Walking bout
