# Supplementary material for: Effects of multidisciplinary inpatient rehabilitation on everyday life physical activity and gait in patients with multiple sclerosis
Source: J Neuroeng Rehabil. 2024 May 28;21:88. doi: 10.1186/s12984-024-01383-0 (PMC11131212; doi:10.1186/s12984-024-01383-0)
Supplement: Supplementary file 1 — Supplementary Material 1. [file 12984_2024_1383_MOESM1_ESM.docx]

**Appendix 1:** **Physical activity metrics**

Table 1. Mapping of physical activity (PA) dimensions into multivariate PA states; each PA state has assigned a numerical code and a color for pattern visualization.

| Type | Duration | Intensity | Multivariate  PA states |
| --- | --- | --- | --- |
| If Type=  Non-locomotion |  | & max($a_{D}$)≤0.2 (very low) | 1 |
|  |  | & 0.2< max($a_{D}$)≤0.5 (low) | 2 |
|  |  | & 0.5< max($a_{D}$)≤1 (medium) | 3 |
|  |  | & 1< max($a_{D}$)≤2 (high) | 4 |
|  |  | & 2< max($a_{D}$) (very high) | 5 |
| If Type=  Locomotion | & duration ≤ 30  (very short) | & cadence≤70 | 6 |
|  |  | & 70<cadence≤90 | 7 |
|  |  | & 90<cadence≤110 | 8 |
|  |  | & 110<cadence≤130 | 9 |
|  |  | & 130<cadence | 10 |
|  | & 30<duration ≤ 120  (short) | & cadence≤70 | 11 |
|  |  | & 70<cadence≤90 | 12 |
|  |  | & 90<cadence≤110 | 13 |
|  |  | & 110<cadence≤130 | 14 |
|  |  | & 130<cadence | 15 |
|  | & 120<duration ≤ 360  (medium) | & cadence≤70 | 16 |
|  |  | & 70<cadence≤90 | 17 |
|  |  | & 90<cadence≤110 | 18 |
|  |  | & 110<cadence≤130 | 19 |
|  |  | & 130<cadence | 20 |
|  | & 360<duration  (long) | & cadence≤70 | 21 |
|  |  | & 70<cadence≤90 | 22 |
|  |  | & 90<cadence≤110 | 23 |
|  |  | & 110<cadence≤130 | 24 |
|  |  | & 130<cadence | 25 |

The sedentary behaviour corresponds to the PA states 1 and 2 combined. The locomotion periods represent all PA states from 6 to 25. Agiovlasitis *et al.,* established the relationship between metabolic equivalent units (METs) and step-rate in persons with MS. A range from 83 to 104 steps/min, depending on the subject’s height, was found to correspond to 3 METs, which is commonly used as the threshold between light to moderate exercise intensities (Agiovlasitis and Motl, 2014). Consequently, we choose 90 steps/min as an appropriate cut-off between light (LPA) and moderate-to-vigorous PA (MVPA). Thus, LPA is defined as the percentage locomotion per day with a cadence lower or equal than 90 steps/min (i.e., PA states 6, 7, 11, 12, 16, 17, 21, 22), and the MVPA as the percentage locomotion per day with a cadence higher than 90 steps/min (i.e., PA states 8, 9, 10, 13, 14, 15, 18, 19, 20, 23, 24, 25). The remaining PA states (i.e., 3, 4, and 5) mainly correspond to short movements such as stepping shorter than a WB, or other foot movements that are not considered as locomotion.

**Appendix 2:** **Linear mixed-effect (LME) model obtained for the self-reported questionnaires and the walking tests assessed during supervised conditions**

Table 2. LME model obtained for the self-reported questionnaires and the walking tests assessed during supervised conditions

| **Responder** | **Predictor** | $\boldsymbol{\gamma}$ | **Estimate** | **\|t\|** | **p** | **95% CI for estimate** | | **Total predict (**$\boldsymbol{R}^{\boldsymbol{2}}$**)** | |
| --- | --- | --- | --- | --- | --- | --- | --- | --- | --- |
| **FSMCt** | intercept | $\gamma_{00}$ | 68.21 |  |  |  |  | | $0.95$ |
|  | rehab | $\gamma_{10}$ | **-4.25** | **-2.68** | **0.009*** | **-7.40** | **-1.10** | |  |
|  | group | $\gamma_{01}$ | -8.26 | -1.59 | 0.116 | -18.60 | 2.08 | |  |
|  | group*rehab | $\gamma_{11}$ | -1.54 | -0.65 | 0.521 | -6.29 | 3.21 | |  |
| **FSMCk** | intercept | $\gamma_{00}$ | 29.50 |  |  |  |  | | $0.96$ |
|  | rehab | $\gamma_{10}$ | -1.58 | -1.73 | 0.087 | -3.40 | 0.24 | |  |
|  | group | $\gamma_{01}$ | -2.66 | -0.83 | 0.410 | -9.04 | 3.72 | |  |
|  | group*rehab | $\gamma_{11}$ | -0.89 | -0.65 | 0.520 | -3.63 | 1.85 | |  |
| **FSMCm** | intercept | $\gamma_{00}$ | 38.71 |  |  |  |  | | $0.93$ |
|  | rehab | $\gamma_{10}$ | **-2.67** | **-3.33** | **0.001*** | **-4.26** | **-1.07** | |  |
|  | group | $\gamma_{01}$ | **-5.60** | **-2.37** | **0.020*** | **-10.32** | **-0.89** | |  |
|  | group*rehab | $\gamma_{11}$ | -0.65 | -0.54 | 0.592 | -3.05 | 1.75 | |  |
| **EQ-VAS** | intercept | $\gamma_{00}$ | 52.88 |  |  |  |  | | $0.84$ |
|  | rehab | $\gamma_{10}$ | **6.42** | **2.50** | **0.014*** | **1.32** | **11.52** | |  |
|  | group | $\gamma_{01}$ | 6.34 | 1.22 | 0.227 | -4.02 | 16.70 | |  |
|  | group*rehab | $\gamma_{11}$ | 5.58 | 1.45 | 0.151 | -2.09 | 13.25 | |  |
| **MSWS-12** | intercept | $\gamma_{00}$ | 82.23 |  |  |  |  | | $0.94$ |
|  | rehab | $\gamma_{10}$ | **-9.41** | **-4.30** | **0.000*** | **-13.76** | **-5.05** | |  |
|  | group | $\gamma_{01}$ | **-16.82** | **-4.49** | **0.000*** | **-24.26** | **-9.37** | |  |
|  | group*rehab | $\gamma_{11}$ | 2.76 | 0.84 | 0.405 | -3.79 | 9.30 | |  |
| **TUG** | intercept | $\gamma_{00}$ | 22.15 |  |  |  |  | | $0.92$ |
|  | rehab | $\gamma_{10}$ | **-4.33** | **-2.46** | **0.016*** | **-7.84** | **-0.83** | |  |
|  | group | $\gamma_{01}$ | **-12.74** | **-3.58** | **0.001*** | **-19.83** | **-5.65** | |  |
|  | group*rehab | $\gamma_{11}$ | 2.59 | 0.99 | 0.325 | -2.62 | 7.80 | |  |
| **10mWT** | intercept | $\gamma_{00}$ | 23.09 |  |  |  |  | | $0.90$ |
|  | rehab | $\gamma_{10}$ | **-6.00** | **-3.42** | **0.001*** | **-9.50** | **-2.51** | |  |
|  | group | $\gamma_{01}$ | **-14.25** | **-4.17** | **0.000*** | **-21.05** | **-7.45** | |  |
|  | group*rehab | $\gamma_{11}$ | 4.13 | 1.58 | 0.117 | -1.06 | 9.33 | |  |
| **2MWT** | intercept | $\gamma_{00}$ | 72.36 |  |  |  |  | | $0.95$ |
|  | rehab | $\gamma_{10}$ | **11.45** | **2.02** | **0.047*** | **0.16** | **22.75** | |  |
|  | group | $\gamma_{01}$ | **65.90** | **6.34** | **0.000*** | **45.21** | **86.59** | |  |
|  | group*rehab | $\gamma_{11}$ | **18.76** | **2.25** | **0.027*** | **2.16** | **35.35** | |  |

Bold values with an asterisk (*) correspond to significant results, indicated by p-value<0.05 and 95% confidence interval (CI) that do not include 0.

**Appendix 3: Mean and standard deviation of the self-reported questionnaires and functional tests**

Table 3. Mean and standard deviation (std) across subjects for the self-reported questionnaires and the walking tests assessed during supervised conditions.

|  | **Mild disability (EDSS < 5)** | | | | **Severe disability (EDSS > 5)** | | | |
| --- | --- | --- | --- | --- | --- | --- | --- | --- |
|  | **Pre** | **Post** | **Δ** | **Δ (%)** | **Pre** | **Post** | **Δ** | **Δ (%)** |
| **FSMCt** | 59.9 (20.3) | 54.2 (16.3) | -5.8 (8.0) | -7.6 (12.2) | 67.5 (14.6) | 63.3 (13.7) | -4.3 (8.1) | -5.6 (11.5) |
| **FSMCm** | 33.1 (9.5) | 29.8 (7.9) | -3.3 (4.3) | -8.4 (13.6) | 38.5 (6.5) | 35.8 (6.5) | -2.7 (3.9) | -6.5 (9.5) |
| **FSMCk** | 26.8 (11.5) | 24.4 (9.2) | -2.5 (4.7) | -5.6 (16.1) | 29.0 (10.0) | 27.4 (9.1) | -1.6 (4.6) | -3.4 (18.1) |
| **EQ-VAS** | 59.2 (15.5) | 71.2 (14.6) | 12.0 (14.6) | 32.7 (46.7) | 52.0 (14.0) | 58.4 (15.7) | 6.4 (11.5) | 15.2 (25.1) |
| **MSWS-12** | 65.4 (20.5) | 58.8 (20.4) | -6.7 (10.9) | -10.6 (21.0) | 81.9 (9.6) | 72.0 (13.4) | -9.9 (11.0) | -12.0 (13.5) |
| **TUG (s)** | 9.4 (3.0) | 7.7 (2.5) | -1.7 (2.8) | -15.4 (22.4) | 21.4 (15.5) | 17.9 (10.2) | -4.3 (11.7) | -12.0 (22.4) |
| **10mWT (s)** | 8.8 (4.0) | 7.0 (1.5) | -1.9 (3.4) | -13.9 (23.0) | 22.4 (14.8) | 16.3 (8.8) | -6.4 (11.3) | -20.0 (25.7) |
| **2MWT (m)** | 138.3 (35.0) | 168.5 (39.8) | 30.2 (36.0) | 27.2 (38.8) | 74.5 (32.4) | 82.0 (37.3) | 11.7 (16.8) | 20.3 (29.0) |
| **Gait velocity**  **10mWT (m/s)** | 1.3 (0.4) | 1.5 (0.3) | 0.2 (0.4) | - | 0.6 (0.3) | 0.8 (0.3) | 0.2 (0.2) | - |
| **Gait velocity**  **2MWT (m/s)** | 1.2 (0.3) | 1.4 (0.3) | 0.3 (0.3) | - | 0.6 (0.3) | 0.7 (0.3) | 0.1 (0.1) | - |

**Appendix 4: Mean and standard deviation of the PA and gait metrics**

Table 4. Mean and std across subjects for the PA and gait parameters before and after the intervention period.

|  |  | **Mild disability (EDSS < 5)** | | **Severe disability (EDSS > 5)** | |
| --- | --- | --- | --- | --- | --- |
|  |  | **Pre**  **mean (std)** | **Post**  **mean (std)** | **Pre**  **mean (std)** | **Post**  **mean (std)** |
| **Sed (%)** | median | 84.85 (3.58) | 83.04 (5.9) | 89.75 (5.77) | 90.58 (5.61) |
|  | max | 91.42 (4.18) | 89.51 (4.75) | 94.20 (4.04) | 94.42 (4.13) |
| **Loc (%)** | median | 13.09 (3.34) | 14.74 (5.13) | 8.54 (5.40) | 7.90 (4.97) |
|  | max | 20.04 (6.56) | 21.65 (5.68) | 12.08 (6.93) | 11.72 (6.91) |
| **LPA (%)** | median | 7.89 (2.91) | 8.36 (3.34) | 7.12 (3.46) | 6.58 (3.12) |
|  | max | 12.19 (3.40) | 13.54 (3.59) | 10.31 (4.51) | 10.05 (4.26) |
| **MVPA (%)** | median | 5.00 (2.96) | 6.02 (4.04) | 1.32 (2.77) | 1.11 (2.18) |
|  | max | 9.29 (6.00) | 10.30 (5.54) | 2.47 (5.37) | 2.21 (3.89) |
| **Hn** | median | 0.255 (0.048) | 0.276 (0.075) | 0.186 (0.074) | 0.174 (0.072) |
|  | max | 0.349 (0.089) | 0.371 (0.077) | 0.239 (0.079) | 0.236 (0.086) |
| **PLZC** | median | 0.114 (0.025) | 0.121 (0.026) | 0.10 (0.033) | 0.095 (0.034) |
|  | max | 0.149 (0.028) | 0.156 (0.028) | 0.134 (0.033) | 0.128 (0.034) |
| **#steps/day** | median | 4771.7 (1353.8) | 5210.9 (2107.6) | 2584.8 (1921) | 2300.3 (1778.1) |
|  | max | 7506.1 (2822.5) | 8324.89  (2689.1) | 3941.01  (3246.4) | 3781.57  (2799.5) |
| **WB time (s)** | mode | 11.34 (2.25) | 11,54 (2.19) | 12.31 (3.00) | 12.71 (2.98) |
|  | pct95% | 63.23 (37.74) | 68,78 (35.24) | 44.43 (16.48) | 45.98 (17.98) |
| **#gait cycle/WB** | mode | 6.6 (1.6) | 8.0 (3.7) | 6.0 (1.2) | 6.5 (1.7) |
|  | pct95% | 47.54 (31.54) | 53.55 (31.82) | 25.63 (14.97) | 25.18 (12.56) |
| **#WB > 30s** | count | 121.3 (60.6) | 119 (50.1) | 68.4 (60.5) | 51.1 (46.2) |
| **Gait speed (m/s)** | mode | 0.7 (0.09) | 0.71 (0.12) | 0.41 (0.16) | 0.40 (0.16) |
|  | pct95% | 1.07 (0.14) | 1.11 (0.16) | 0.62 (0.19) | 0.62 (0.19) |
| **Cadence (steps/min)** | mode | 95.8 (6.2) | 96.7 (8.1) | 80.1 (14.2) | 79.58 (14.56) |
|  | pct95% | 113.59 (8.33) | 115.71 (10.18) | 108.75 (22.87) | 108.71 (14.72) |
| **Stride length (m)** | mode | 0.91 (0.09) | 0.92 (0.11) | 0.63 (0.18) | 0.61 (0.18) |
|  | pct95% | 1.24 (0.12) | 1.27 (0.13) | 0.87 (0.17) | 0.91 (0.20) |
| **Gait cycle time (s)** | mode | 1.28 (0.07) | 1.27 (0.11) | 1.59 (0.36) | 1.59 (0.36) |
|  | pct95% | 1.94 (0.14) | 1.92 (0.15) | 2.32 (0.49) | 2.35 (0.5) |
